# Seroepidemiology of *Klebsiella pneumoniae *colonizing the intestinal tract of healthy chinese and overseas chinese adults in Asian countries

**DOI:** 10.1186/1471-2180-12-13

**Published:** 2012-01-19

**Authors:** Yi-Tsung Lin, L Kristopher Siu, Jung-Chung Lin, Te-Li Chen, Chih-Peng Tseng, Kuo-Ming Yeh, Feng-Yee Chang, Chang-Phone Fung

**Affiliations:** 1 Division of Infectious Diseases, Department of Medicine, Taipei Veterans General Hospital, Taipei, Taiwan; 2 Institute of Clinical Medicine, School of Medicine, National Yang Ming University, Taipei, Taiwan; 3 Division of Infectious Diseases, National Health Research Institutes, Maoli, Taiwan; 4 Division of Infectious Diseases and Tropical Medicine, Department of Internal Medicine, Tri-Service General Hospital, and National Defense Medical Center, Taipei, Taiwan; 5 Centers for Disease Control, Taipei, Taiwan

## Abstract

**Background:**

Capsular serotypes K1 and K2 of *Klebsiella pneumoniae *are thought to the major virulence determinants responsible for liver abscess. The intestine is one of the major reservoirs of *K. pneumoniae*, and epidemiological studies have suggested that the majority of *K. pneumoniae *infections are preceded by colonization of the gastrointestinal tract. The possibility of fecal-oral transmission in liver abscess has been raised on the basis of molecular typing of isolates. Data on the serotype distribution of *K. pneumoniae *in stool samples from healthy individuals has not been previously reported. This study investigated the seroepidemiology of *K. pneumoniae *isolates from the intestinal tract of healthy Chinese in Asian countries. Stool specimens from healthy adult Chinese residents of Taiwan, Japan, Hong Kong, China, Thailand, Malaysia, Singapore, and Vietnam were collected from August 2004 to August 2010 for analysis.

**Results:**

Serotypes K1/K2 accounted for 9.8% of all *K. pneumoniae *isolates from stools in all countries. There was no significant difference in the prevalence of K1/K2 isolates among the countries excluding Thailand and Vietnam. The antimicrobial susceptibility pattern was nearly the same in *K. pneumoniae *isolates. The result of pulsed-field gel electrophoresis revealed no major clonal cluster of serotype K1 isolates.

**Conclusions:**

The result showed that Chinese ethnicity itself might be a major factor predisposing to intestinal colonization by serotype K1/K2 *K. pneumoniae *isolates. The prevalent serotype K1/K2 isolates may partially correspond to the prevalence of *K. pneumoniae *liver abscess in Asian countries.

## Background

*Klebsiella pneumoniae *is responsible for a wide spectrum of clinical syndromes, including purulent infections, urinary tract infections, pneumonia, bacteremia, septicemia, and meningitis [1]. In the past three decades, *K. pneumoniae *has emerged as the single leading cause of pyogenic liver abscess in East Asian countries, especially in Taiwan [2-7]. An invasive syndrome of liver abscess complicated by meningitis, endophthalmitis or other metastatic suppurative foci has been reported, and capsular serotypes K1 and K2 of *K. pneumoniae *are thought to the major virulence determinants responsible for this syndrome [3,6,8]. In an analysis of *K. pneumoniae *liver abscess from two hospitals in New York by Rahimian et al. [9], 78.3% of patients were of Asian origin. These findings raise the possibility that genetic susceptibility to or geographic distribution patterns of virulent *K. pneumoniae *subtypes may play important roles [10].

The intestine is one of the major reservoirs of *K. pneumoniae*, and epidemiological studies have suggested that the majority of *K. pneumoniae *infections are preceded by colonization of the gastrointestinal tract [11]. The possibility of fecal-oral transmission has been raised on the basis of molecular typing of isolates from siblings, family members, and the environment in one study from Taiwan [12]. One recent study from Japan has demonstrated the familial spread of a virulent clone of *K. pneumoniae *causing primary liver abscess, and has provided evidence that virulent clones of *K. pneumoniae *have colonized family members for at least 2 years [13]. However, data on the serotype distribution of *K. pneumoniae *in stool samples from healthy individuals has not been previously reported.

To explore the ethnicity and geographical question regarding the serotype distribution of *K. pneumoniae *from fecal isolates in different countries, we focused on the same population but in different countries. Therefore, this study investigated the seroepidemiology of *K. pneumoniae *colonizing the intestinal tract, using stool specimens from healthy Chinese adults in different Asian countries.

## Results

### Rate of *K. pneumoniae *isolation from stool specimens

During the study period, a total of 592 (62.1%) *K. pneumoniae *strains were isolated from 954 collected stool specimens. The isolation rate was highest in Malaysia (64/73, 87.7%), followed by Taiwan (150/200, 75%) and Singapore (47/77, 61.1%). The isolation rate was lowest in Japan (6/32, 18.8%) (Table 1).

**Table 1:** Isolation rates of *K. pneumoniae *from stool specimens in healthy adult Chinese and overseas Chinese residents of Asian countries

Country/region	No. of stool samples	No. (%) of isolates
Taiwan	200	150 (75.0)
China	221	128 (57.9)
Hong Kong	85	50 (58.8)
Singapore	77	47 (61.1)
Malaysia	73	64 (87.7)
Thailand	208	123 (52.9)
Japan	32	6 (18.8)
Vietnam	58	24 (41.3)

### Seroepidemiology

Antisera of the recognized 77 serotypes (designated K1-K74 and K80-K82) were used to analyze the isolates. Table 2 shows the distribution of serotypes among the 592 *K. pneumoniae *isolates from stool specimens of healthy Chinese and overseas Chinese adults in different Asian countries. Table 3 shows the distribution of serotypes K1/K2 isolates in different countries. Serotypes K1/K2 isolates accounted for 9.8% of all *K. pneumoniae *strains in all countries. Compared with other countries, Taiwan did not have a significantly higher prevalence of serotypes K1/K2 *K. pneumoniae *(11.3% vs. 9.3%, *p *= 0.46). When excluding Thailand and Vietnam, the prevalence of K1/K2 isolates did not differ among the countries (*p *= 0.98).

**Table 2:** Distribution of serotypes among 592 *K. pneumoniae *isolates from stool specimens of healthy Chinese and overseas Chinese adults in Asian countries

	No. of isolates							
Serotype	Taiwan	China	Hong Kong	Singapore	Malaysia	Thailand	Japan	Vietnam
K1	11	9	5	5	8	0	1	0
K2	6	6	1	2	1	3	0	0
K3	0	4	2	0	1	3	0	0
K4	0	0	1	0	0	2	0	0
K5	0	0	0	1	0	2	0	0
K6	0	0	0	0	0	1	0	0
K7	0	3	2	1	2	1	0	0
K8	0	0	0	0	0	1	0	0
K9	0	0	0	0	0	1	0	0
K10	0	0	0	0	1	0	0	0
K11	0	1	3	1	2	6	0	0
K12	1	0	0	0	1	0	0	0
K13	0	0	0	0	1	0	0	0
K14	0	5	0	1	0	2	0	0
K16	0	3	0	1	2	2	0	0
K17	1	1	0	1	0	1	0	0
K18	0	0	0	0	0	1	0	0
K19	0	2	0	0	1	4	10	0
K20	0	0	0	0	0	0	0	1
K22	1	1	2	1	0	2	0	0
K23	0	1	0	0	0	3	0	0
K24	1	3	1	1	0	1	0	0
K25	0	0	0	1	0	1	0	0
K26	0	0	0	0	0	1	0	0
K27	5	1	0	0	0	1	0	0
K28	9	1	0	0	0	0	0	0
K29	0	1	1	0	1	0	0	0
K30	1	2	1	1	1	0	0	0
K31	0	5	0	1	2	3	0	0
K32	0	0	0	0	1	2	0	0
K33	0	1	0	0	2	4	0	0
K34	0	0	0	0	1	0	0	0
K35	0	5	1	1	1	2	0	0
K36	0	2	1	1	0	0	0	0
K37	1	0	0	0	0	0	0	0
K38	2	0	0	0	1	0	1	0
K39	7	0	1	0	0	1	0	0
K40	0	0	0	0	1	0	0	0
K41	0	1	0	0	0	0	0	0
K42	0	1	1	0	1	2	0	0
K43	0	0	0	0	0	1	0	0
K44	0	0	0	2	0	2	0	0
K45	0	0	1	0	0	0	0	0
K46	0	3	0	1	1	1	0	0
K47	0	6	1	1	0	2	0	0
K48	0	1	1	1	0	1	0	0
K49	0	1	0	1	0	1	0	0
K50	1	0	0	0	0	0	0	0
K51	0	1	0	2	1	2	0	0
K52	3	0	1	0	0	0	0	0
K53	0	8	0	0	1	1	0	0
K54	2	5	2	0	1	3	0	1
K55	0	4	1	0	1	7	0	0
K56	0	0	1	0	0	2	1	0
K57	3	1	1	0	0	3	0	1
K58	0	1	0	1	0	3	0	0
K59	0	1	1	0	1	0	0	0
K60	0	0	2	0	0	2	0	0
K61	0	0	0	0	0	2	0	0
K62	0	1	0	2	2	1	0	0
K63	2	1	0	1	0	1	0	0
K64	0	0	0	0	1	0	0	0
K65	0	1	0	0	0	0	0	0
K66	1	1	0	0	0	0	0	0
K67	11	0	0	0	1	0	0	0
K68	0	1	0	0	0	1	0	0
K69	0	0	0	0	0	0	1	0
K70	0	3	0	0	0	3	0	0
K71	0	0	0	1	0	0	0	0
K72	5	0	0	0	1	2	0	0
K74	0	0	0	0	1	0	0	0
K79	0	1	1	0	1	3	0	0
K80	0	0	0	0	1	0	0	0
K81	0	0	0	1	2	5	1	0
K82	0	0	0	1	2	8	0	0
Non typable	76	28	16	12	14	13	0	21
Total	150	128	50	47	64	123	6	24

**Table 3:** Distribution of serotypes K1/K2 *K. pneumoniae *isolates from stool specimens of healthy Chinese and overseas Chinese adults in Asian countries

	Taiwan	China	Hong Kong	Singapore	Malaysia	Thailand	Japan	Vietnam
	n = 150	n = 128	n = 50	n = 47	n = 64	n = 123	n = 6	n = 24
Serotype K1	11 (7.3)	9 (7)	5 (10)	5 (10.6)	8 (12.5)	0 (0)	1 (16.7)	0 (0)
Serotype K2	6 (4)	6 (4.7)	1 (2)	2 (4.3)	1 (1.6)	3 (2.7)	0 (0)	0 (0)

Data are presented as no. (%) of isolates

### Antimicrobial susceptibility testing

We randomly and proportionally selected 100 serotypable isolates from different countries for antimicrobial susceptibility testing. The antimicrobial susceptibility pattern was the same in all 97 *K. pneumoniae *isolates, with uniform resistance to ampicillin and susceptibility to all cephalosporins and aminoglycosides. Serotypes K1/K2 and non-K1/K2 had the same antimicrobial susceptibility pattern (data not shown). Two isolates, including one serotype K1 isolate from Taiwan and one non-K1/K2 serotype from Thailand, were resistant to ampicillin and cefazolin but susceptible to other cephalosporins and aminoglycosides. One serotype K1 isolate from Taiwan was resistant to ampicillin, cefazolin, and amikaicin, but susceptible to other cephalosporins. No extended spectrum β-lactamase isolate was detected during this study.

### Pulsed-field gel electrophoresis (PFGE) and screening for CC23 representatives by detection of *allS *by PCR among K1 isolates

**Figure 1:**
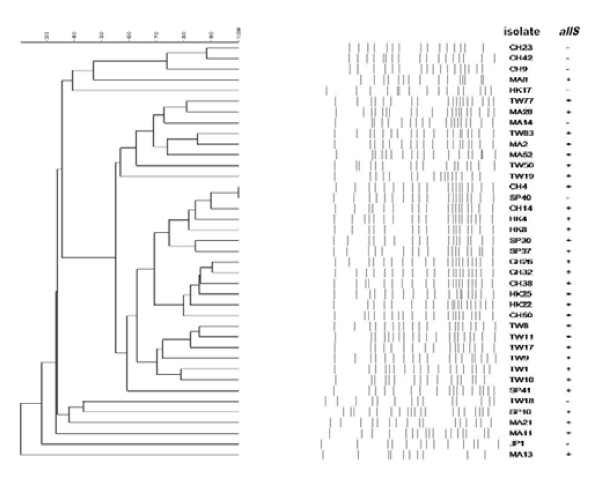
**Dendrogram comparing PFGE profile of *K. pneumoniae *serotype K1 isolates together with the results of *allS *detected by PCR**. No major clonal cluster of serotype K1 *K. pneumoniae *isolates was found. TW, Taiwan; CH, China; SP, Singapore; MA, Malaysia; HK, Hong Kong; JP, Japan.

**Figure 2:**
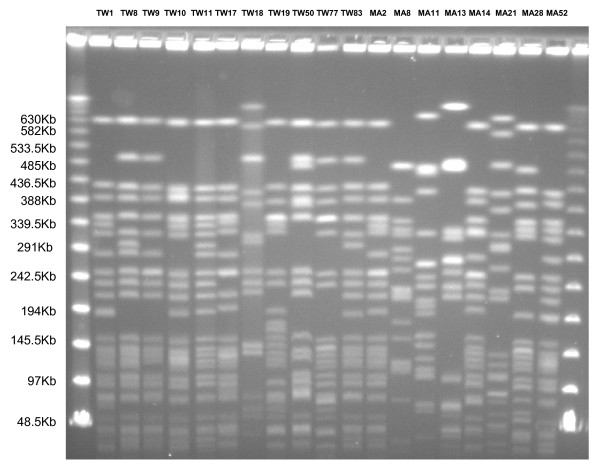
**PFGE profile of *K. pneumoniae *serotype K1 isolates from Taiwan and Malaysia**. TW, Taiwan; MA, Malaysia.

**Figure 3:**
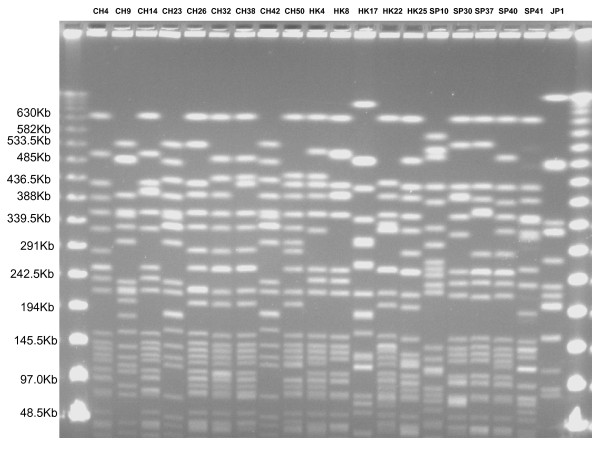
**PFGE profile of *K. pneumoniae *serotype K1 isolates from China, Hong Kong, Singapore and Japan**. CH, China; HK, Hong Kong; SP, Singapore; JP, Japan.

PFGE and detection of *allS *gene by PCR among serotype K1 isolates are shown in Figure 1. The original PFGE profiles are shown in Figure 2 and Figure 3. 31 (79.5%) of the K1 isolates carried *allS *gene. No major cluster was found among serotype K1 isolates from Asian countries, using previously described criteria [3].

## Discussion

The K1 serotype of *K. pneumoniae *was uncommon among clinical isolates before the 1990s [14]. However, K1 serotype infection has been more widespread in Asian countries despite a recently reported increasing role of *K. pneumoniae *in liver abscess in the United States [15,16]. The reason for the epidemiological changes and global differences observed remains unexplained. In this study focusing on Chinese in different Asian regions, a substantial proportion of serotype K1/K2 *K. pneumoniae *strains colonizing the intestine, except for Thailand and Vietnam, suggest that Chinese ethnicity itself might be a major factor predisposing to intestinal colonization by these strains. It also corresponds to the prevalence of liver abscess in Asian countries. The differences in socioeconomic factors, dietary practices, environmental exposure, living conditions, and the use of antimicrobial agents might also have a potential role for the geographic differences in seroepidemiology among *K. pneumoniae *isolates.

In our previous study in Taiwan, 77.6% of *K. pneumoniae *liver abscesses were caused by serotype K1 or K2 isolates [3]. A previous study has found that *K. pneumoniae *isolates from patients with liver abscesses in Singapore and Taiwan have similar characteristics, such as genomic heterogeneity and prevalence of virulence factors [6]. The prevalence of serotypes K1/K2 *K. pneumoniae *colonizing the intestinal tract in Taiwan is similar to that in Singapore. The prevalence of serotype K1/K2 *K. pneumoniae *isolates colonizing the intestine may contribute to invasive liver abscess syndrome in Taiwan and Singapore.

In Hong Kong, serotype K1 isolates from liver abscess specimens were studied, but the associated clinical details of the patients were not available [17]. A recent study from Japan has reported familial spread of a K1 clone of *K. pneumoniae *causing primary liver abscess [13]. In another study from Malaysia [18], *K. pneumoniae *rarely caused liver abscess and isolates were not serotyped [18]. In a recent study in China, *K. pneumoniae *was the prevalent pathogen in liver abscess but the serotypes of isolates were unavailable [19]. Further research focusing on serotype of *K. pneumoniae *isolates in these countries might clarify the relation between colonization and infection. *K. pneumoniae*-associated liver abscess caused by serotype K1 has never been reported in Thailand or Vietnam. Interestingly, we did not find any serotype K1 *K. pneumoniae *isolate from stools in the two countries.

In the present study, there was no major clonal cluster of serotype K1 isolates in Asian countries. Although one previous study of the molecular epidemiology of liver abscess in Taiwan identified a major cluster of *K. pneumoniae *isolates causing liver abscess [20], subsequent studies with the methods of ribotyping and PFGE have shown that *K. pneumoniae*-related liver abscesses are not caused by a clonally-spread strain [3,21,22]. Another study has further demonstrated that *K. pneumoniae *isolates causing liver abscess are not clonal in either Singapore or Taiwan [6]. Turton et al. firstly reported that the prevalence of strain ST23 in liver abscesses in Taiwan was high and that the strains were clonally related [17]. In the current study, we screened for strain CC23 representatives by detection of *allS *by PCR [23] and found that isolates carrying *allS *were also predominant in serotype K1 *K. pneumoniae *present in healthy adult stools. However, isolates carrying *allS *from stools were not related by PFGE, indicating that a geographic difference might account for the diversity.

An important limitation of this study was the lack of data regarding Chinese residents in Korea. Invasive liver abscess caused by *K. pneumoniae *K1 serotype has been emerging in Korea [5,24]. A further study of the serotype and genetic relatedness of *K. pneumoniae *isolates colonizing the intestine in Korea may elucidate the epidemiology of emerging disease caused by K1 *K. pneumoniae *in Asia. Future investigation of *K. pneumoniae *from stools in Western countries is also needed to delineate the global epidemiology and the relation with *K. pneumoniae *liver abscess.

## Conclusions

This is believed to be the first report to demonstrate the seroepidemiology of *K. pneumoniae *colonizing the intestinal tract of Chinese healthy adults in Asian countries. Serotype K1/K2 comprised 9.8% of the *K. pneumoniae *strains in this study. The antimicrobial susceptibility pattern was nearly the same in *K. pneumoniae *isolates, with uniform resistance to ampicillin and susceptibility to all cephalosporins and aminoglycosides. There was no significant difference in the prevalence of K1/K2 isolates among the countries, excluding Thailand and Vietnam. No major clonal cluster was found among serotype K1 isolates in Asian countries. Chinese ethnicity itself might be a major factor predisposing to intestinal colonization by these strains. The prevalent serotype K1/K2 isolates may partially correspond to the prevalence of *K. pneumoniae *liver abscess in Asian countries.

## Methods

### Sample collection and bacterial identification

In this study, stool specimens from healthy adult Chinese residents of Taiwan, Hong Kong and China, and overseas Chinese in Japan, Thailand, Malaysia, Singapore and Vietnam were collected from August 2004 to August 2010. A total of 954 healthy adult volunteers (age > 20 years old) were invited to participate and provide stool samples for the study. They had no history of travel abroad, no gastrointestinal disease, and no hospital admission in the past year. None of them had been given any antibiotics during the 3 months before collection of the stool samples.

Stool samples were collected and placed in Cary-Blair transport medium, transported to a microbiology laboratory and inoculated on MacConkey agar plates and *K. pneumoniae *selective medium for the isolation of *K. pneumoniae*. The API 20E system (Bio-Merieux, Marcy I'Etoile, France) was used to identify isolates of *K. pneumoniae*. During the study period, the participants gave oral consent and voluntarily provided their stool samples for analysis of *K. pneumoniae *after stool routine procedures in the physical check-up. It was not possible to identify the patients from the data; therefore, the study was considered exempt from review by the Institutional Review Board of Taipei Veterans General Hospital.

### Serotyping and PCR

All isolates were serotyped by a countercurrent immunoelectrophoresis method [25]. Antisera were kindly provided by the Laboratory of HealthCare Associated Infection, Centre for Infections, Health Protection Agency, London. *K. pneumoniae *ATCC9997 (K2) was used as a control strain. K1 and K2 isolates were confirmed by PCR as described previously [26]. All K1 isolates were screened for CC23 representatives by detection of *allS *by PCR as described previously [23].

### Antimicrobial susceptibility testing

Susceptibility to antimicrobial agents was determined by the disc diffusion method on Mueller-Hinton agar medium (BBL Microbiological Systems, Cockeysville, MD, USA). The antibiotics tested were ampicillin (10 μg), cefazolin (30 μg), cefonicid (30 μg), cefotaxime (30 μg), ceftriaxone (30 μg), cefoperazone (75 μg), ceftazidime (30 μg), gentamicin (10 μg), and amikacin (30 μg). Interpretations were performed according to Clinical and Laboratory Standards Institute guidelines [27].

### PFGE

Total DNA was prepared, and PFGE was performed as described previously [3]. The restriction enzyme *Xba*I (New England Biolabs, Beverly, MA, USA) was used. Restriction fragments were separated by PFGE in 1% agarose gel (Bio-Rad, Hercules, CA, USA) in 0.5 × Tris-boric acid-EDTA buffer using a Bio-Rad CHEF-Mapper apparatus (Bio-Rad Laboratories, Richmond, CA, USA). Gels were stained with ethidium bromide and photographed under UV light. Dendrograms showing percentage similarity were developed with Molecular Analyst Fingerprinting Software (Bio-Rad Laboratories, Hercules, CA, USA) and compared using the UPGMA clustering method. A similarity coefficient > 80% was selected to define a major cluster.

### Statistical analysis

Contingency data were analyzed by two-tailed *χ*^2 ^test or Fisher's exact test as appropriate. A *p *value < 0.05 was considered to be statistically significant, and all probabilities were two-tailed. All statistical analyses were performed with SPSS for Windows version 15.0 (SPSS, Chicago, IL, USA).

## Conflicts of interests

The authors declare that they have no competing interests.

## Authors' contributions

YTL participated in the study design, carried out laboratory work, analyzed the data, and drafted the manuscript. LKS participated in the study design, collected the specimens, carried out laboratory work, and analyzed the data. JCL participated in the study design, carried out laboratory work, and analyzed the data. TLC conceived the study, collected the specimens, and edited the manuscript. CPT, KMY and FYC conceived the study and edited the manuscript. CPF conceived the study, participated in its design and coordination, collected the specimens, analyzed the data, edited the manuscript, and received the majority of funding needed to complete the research. All authors have read and approved the final manuscript.
